# Successful pregnancy via in vitro fertilization in a primary infertile woman with primary lymphoma of the fallopian tube after surgery: A case report and literature review

**DOI:** 10.1097/MD.0000000000029353

**Published:** 2022-07-29

**Authors:** Jie Zhang, Zhen Hou, Jie Huang, Wei Xu, Cong Wang, Xiang Ma, Nan Lu, Jiayin Liu, Yundong Mao, Yi Qian

**Affiliations:** a State Key Laboratory of Reproductive Medicine, Clinical Center of Reproductive Medicine, First Affiliated Hospital, Nanjing Medical University, Nanjing, Jiangsu, China; b Hematology Department of the First Affiliated Hospital, Nanjing Medical University, Nanjing, Jiangsu, China; c Pathology department of the First Affiliated Hospital, Nanjing Medical University, Nanjing, Jiangsu, China.

**Keywords:** B-Cell, fallopian tube, infertility, lymphoma, marginal zone

## Abstract

**Rationale:**

Primary extranodal marginal zone B-cell lymphomas of the fallopian tube is extremely rare. It is a great challenge for fertility and gynecology doctors to manage such cases and also fulfil the reproductive demands of these young women.

**Patient concerns:**

A 30-year-old woman consulted for a 5-year primary infertility.

**Diagnosis:**

According to the Ann Arbor staging system, a stage IE extranodal marginal zone B-cell lymphoma of mucosa-associated lymphoid tissue lymphoma was diagnosed for this patient based on tumor pathology, bone marrow biopsy, hysteroscopy and whole-body positron emission tomography imaging. She also had endometriosis based on laparoscopy.

**Interventions:**

The patient underwent a laparoscopic bilateral salpingostomy without additional surgery or radiotherapy and chemotherapy for fertility preservation, and received 5 months of long-acting gonadotropin releasing hormone agonist treatment for endometriosis.

**Outcomes:**

Two years after the surgery, the patient delivered a healthy neonate through in vitro fertilization-embryo transfer procedures. The patient is now 3 years post-op and 1 year post-delivery, long-term follow-up suggested that the patient remained cancer-free up till now.

**Lessons:**

More care should be taken when the newly diagnosed mass is combined with a rather high serum CA-125 level. Although endometriosis and ovarian cancer are more common, lymphoma cannot be ruled out.

## 1. Introduction

Extranodal marginal zone B-cell lymphoma (MZBL) of mucosa-associated lymphoid tissue (MALT)-type is a B-cell lymphoma originated from mucosa associated lymphoid tissue. Gastric mucosa is the most common location of the MALT lymphoma, accounting for over 50% of all cases. Beyond that, lung, thyroid, salivary gland, and thymus are other common sites.^[1]^ The primary lymphomas of the female genital tract are extremely rare, especially in the fallopian tube. To the best of our knowledge, only 6 cases of primary fallopian tube lymphomas were ever reported,^[2–7]^ and all of those women did not have fertility requirements. Thus, this is the first reported case of an infertile woman with MZBL of MALT-type involving the fallopian tube. The patient provided signed informed consent, and Institutional Review Board approval was obtained (No. 2019C002).

## 2. Case presentation

A 30-year-old woman consulted for a 5-year primary infertility. She had slight dyspareunia, but no dysmenorrhea, pelvic pain, fever, and wasting. When she was 21 years old, she underwent laparoscopic right ovarian cyst enucleation due to serous cystadenoma of the right ovary, and the cystadenoma was about 15 cm*16 cm*10 cm in size. She had a 20-year history of hepatitis B infection and had received treatment, after which the hepatitis B DNA dropping to a normal range. The gynecological examination results included poor mobility and tenderness of the uterus, as well as tenderness of right adnexal and uterosacral ligament. Hysterosalpingography indicated bilateral distal tubal obstruction and mild hydrocephalus. Three-dimensional ultrasound indicated the presence of mild adenomyosis and a hypoechoic mass in the left ovary, which was about 8 mm*8 mm in size. Tumor markers: CA-125 658.7 U/ml. Considering the history of infertility and dyspareunia, tenderness of uterosacral ligament, elevated serum CA-125 level and ultrasound performance, endometriosis was highly suspected and surgical exploration was recommended. The patient and her husband refused and required conservative medication due to concerns regarding a second abdominal surgery. Then the patient received 4 months of long-acting gonadotropin releasing hormone agonist (GnRH-a) treatment under suspicion of endometriosis and adenomyosis. Three-dimensional ultrasound of reexamination indicated hypoechoic sign outside the bilateral ovaries, which was considered as bilateral pyosalpinx. Tumor markers levels reexamination showed CA-125 593.5 U/ml, AFP, CA-50, CA-199 and HE4 all in normal range. Laboratory tests including total blood count, serum C-reactive protein, procalcitonin levels, biochemistry test and coagulation function, were all normal.

**Figure 1.**
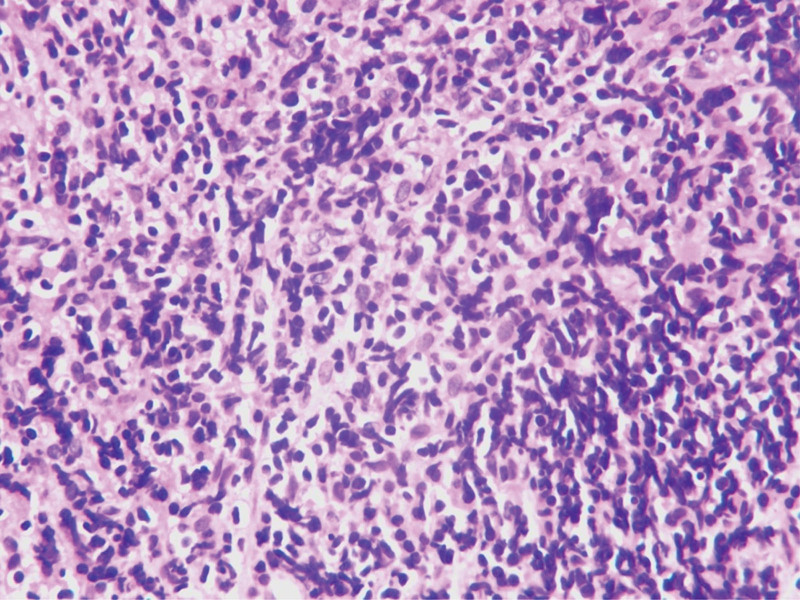
Hematoxylin and eosin staining; magnification, ×400.

**Figure 2.**
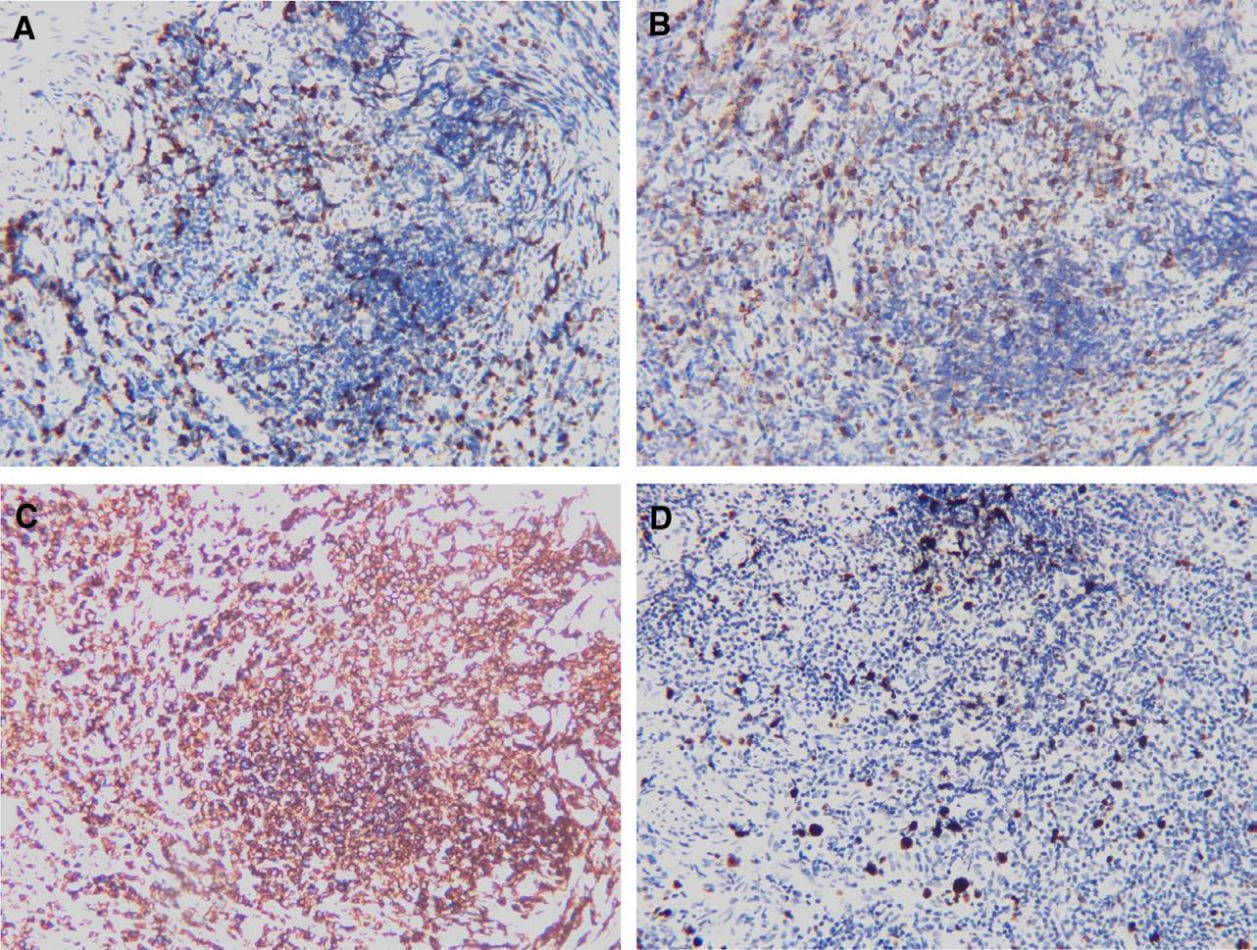
Immunohistochemical examination were positive for CD3(A), CD5(B), CD20(C), Ki67(D)staining; magnification, ×200 in A, B, C, D.

Due to the failure of conservative treatment, a laparoscopic bilateral salpingostomy was performed under the presumptive diagnosis of bilateral pyosalpinx. During the operation, findings included thickening, twisting bilateral fallopian tubes with atresia fimbria, scattered endometriosis lesions and mild adhesion. Endometriosis lesions were removed and adhesions were dissolved. Gross images showing: thin pus in the right fallopian tube and papillary process appeared in the lumen of the left fallopian tube. General bacterial culture of the right fallopian tube pus was negative. The pathological results showed that bilateral fallopian tubes were infiltrated with abundant inflammatory cells, and the lymphoid tissue was highly proliferated with significant follicular implantation and plasma-cell differentiation (Fig. 1), which required further immunohistochemistry and gene analysis. As expected, clonal rearrangements of the immunoglobulin (lg) heavy chain locus rearrangements were positive. Immunohistology demonstrated: (diseased cell) CD20 (+), Pax-5 (+), CD10 (−), Bcl-6 (−), Bcl-2 (+), Ki-67 (+), CD43 (−), Mum1 (partly +), CK-pan (−), CD38 (partly +), Igκ (++), Igλ (+), Cyclin D1 (−); (Residual follicles and background cells) CD21 (+), CD23 (+), CD3 (+), CD5 (+) (Fig. 2A-D). The findings were consistent with the features of extranodal MZBL of MALT lymphoma. A month after surgery, serum CA-125 concentrations decreased to 37.7 U/ml.

Following the diagnosis of lymphoma, bone marrow biopsy and detection of minimal residual lesions in bone marrow were performed, which were both negative. The patient also underwent hysteroscopy, endometrial biopsy and whole-body positron emission tomography imaging, and these findings did not show disease involvement with other sites. The disease was clinically staged as IE according to the Ann Arbor staging system modified for extranodal lymphoma. During multi-disciplinary consultation, hematologists proposed that localized MALT lymphoma is usually treated with local radiotherapy, however, this would greatly impair the reproductive system. Because the patient had a strong desire for fertility, hematologists recommended the delay of radiotherapy and closely observation with gynecological ultrasound for the preservation of fertility. Therefore, the patient was treated with GnRH-a for 5 months before receiving in vitro fertilization-embryo transfer without additional radiotherapy. Finally, the patient successfully achieved a live birth of a healthy neonate after 2 cycles of IVF treatment. After 3 years of follow-up, she is still in good condition.

## 3. Discussion

Primary lymphoma of the female genital tract (PLFGT) is a very rare entity, accounting for only 2% of all extranodal primary lymphoma. The most common sites of PLFGT are the ovary and cervix, and involvement of the fallopian tubes is extremely rare with only 6 cases reported (Table 1).^[1–6]^

**Table 1:** Articles about primary tubal lymphoma.

Study	Age	Symptom	Sites	Gross	Pathology	Therapy	Stage*	Follow-up
Noack, 2002^[2]^	34	Pelvic pain and cystic solid mass of the left fallopian tube	Left fallopian tube	Distorted right fallopian tube; left fallopian tube was distended and filled with pus.	Extranodal MZBL	Salpingectomy	IE	1 yr, no evidence of recurrence
Gaffan, 2004^[3]^	51	Systemic upset and a pelvic mass	Both fallopian tubes	Inflamed oedematous fallopian tubes	Peripheral T-cell lymphoma	Bilateral salpingectomy and appendicectomy+ CHOP-M	IIE	8 yrs, no evidence of recurrence
Alduaij, 2010^[7]^	68	Incidentally discovered in specimen for endometrial adenocarcinoma	Left fallopian tube	Left fallopian tube showed a 1 cm nodule encasing the lumen.	Follicular lymphoma	Hysterectomy and bilateral salpingooophorectomy	IE	13 mo, no evidence of recurrence
Cho, 2011^[4]^	43	Menorrhagia and dysmenorrhea	Right fallopian tube	Right fallopian tube with slight inflammation	Extranodal MZBL	Right salpingectomy, and hysterectomy	IE	1 yr, no evidence of recurrence
Nezhat, 2013^[5]^	47	Severe dysmenorrhea	Right ovary, fallopian tube, appendix, and right pelvic sidewall	Thick adhesions of both ovaries and fallopian tubes to the pelvic sidewalls	Extranodal MZBL	Removal of the uterus, cervix, bilateral ovaries, appendix	IE	1 yr, no evidence of recurrence
Ye Zhou, 2021^[6]^	52	Intermittent lower abdominal pain and pelvic mass	Right fallopian tube	Thickened and hard right fallopian tube	DLBCL	Hysterectomy and bilateral salpingo-oophorectomy+ R-CHOP	IE	1 yr, no evidence of recurrence

Clinical diagnosis of primary lymphoma of the fallopian tube is difficult. Based on prior reports and our case,^[2–7]^ its clinical manifestations are unremarkable and unspecific, such as dysmenorrhea and pelvic pain, other than the typical signs of lymphoma such as swelling of surface lymph nodes, fever, night sweats, and weight loss. Localized symptoms such as dysmenorrhea and pelvic pain may also be associated with pelvic inflammatory disease, endometriosis, and adenomyosis. These common and unspecific symptoms tend to obscure the diagnosis, while common and benign diseases will be given priority consideration to in young patients, such as endometriosis or pelvic inflammatory diseases. Ultrasound often mostly indicates an adnexal mass, which was suspected to be salpingitis. Due to the lack of characteristic manifestations, lymphoma of the female genital tract always needs to be differentiated from common diseases in this site. The Ann Arbor lymphoma classification is the current staging system for patients with non-Hodgkin lymphoma (NHL).

Extranodal marginal zone B-cell lymphomas are extremely rare in this location. The diagnosis and treatment still lack unified standard and is influenced by the general principals of extranodal MZBL of MALT-type or NHL treatment. Overall, it has a slow clinical course and good prognosis,^[8]^ so the treatment options could be individualized. According to the 2021 National Comprehensive Cancer Network Guideline for non-gastrointestinal extranodal MZBL, radiotherapy is preferred in patients with stage I and II. When the disease is localized, the patient could also be treated with only surgery, as the guideline emphasizes that surgical excision for adequate diagnosis may be appropriate treatment for the disease. In our case, lymphoma was found incidentally during surgery and final histology showed no invasion of the surgical margins. In case of negative surgical margins, the guideline suggests that the following management after surgery could be continuous observation of the patient’s condition. So, our patient did not receive additional chemotherapy or radiotherapy, and remained disease free for more than 3 years. Another similar case was described in a 34-year-old woman with a primary tubal lymphoma of the MALT-type, she was disease free 1 year after salpingectomy without additional chemotherapy or radiotherapy.^[2]^

This type of the lymphoma often occurs in patients with immune or inflammatory diseases. In the female reproductive organs where there is almost no lymphoid tissue, the lymphocytes in the blood stream are the basis of malignant lymphoma. Chronic inflammation activates NF-кB pathway mediated B cell cloning. In our patient, laparoscopic exploration showed a distended right fallopian tube filled with pus but without culture proven infection. It is unclear whether lymphoma appeared before or after salpingitis in this patient. Our patient also had endometriosis, which is a common and chronic inflammatory disorder. Small implants of peritoneal endometriosis can cause acute inflammation, and the immune surveillance systems are also impaired with a decreased clearance ability of natural killer cells and T-lymphocytes.^[9]^ In fact, endometriosis is reportedly associated with higher risks of ovarian cancer, breast cancer, and hematopoietic malignancies.^[10]^ Nezhat et al^[5]^ also reported a case of a MZBL accompanied by endometriosis.

It is reported that serum CA-125 levels are elevated in 40% to 70% of patients with NHL.^[11]^ Our patient also had an elevated serum CA-125 level, which was initially considered a sign of endometriosis. After 4 months of GnRH-a treatment, CA-125 level was still high. However, CA-125 level quickly decreased to normal after laparoscopy, indicating that lymphoma was mainly responsible for CA-125 elevation, other than endometriosis. It is reported that CA-125 levels correlate with NHL activity, treatment response and survival rate.^[12]^ It will decrease during disease remission, and return to elevated levels in disease recurrence. Over 50% primary fallopian tube carcinoma also have elevated serum CA-125 levels,^[13]^ so the identification of primary fallopian tube carcinoma and PLFGT can only depend on histological diagnosis.

According to the guideline, patients with stage III and IV or relapsing or aggressive NHL may benefit from radiotherapy and/or chemotherapy. Preservation of ovarian function should be taken into account under this circumstance. The human oocyte is very sensitive to radiation, and a dose of less than 2 Gy is estimated to be sufficient to destroy 50% of primordial follicles.^[14]^ Alkylating agents are considered to be the most toxic ones, particularly cyclophosphamide, which can lead to iatrogenic premature ovarian insufficiency, and loss of fertility.^[15]^ Unfortunately, CHOP (cyclophosphamide, doxorubicin, vincristine, and prednisolone) is the most standard regimen for NHL treatment, which contains alkylating agents. However, there is a surprisingly low incidence of long-term gonadal damage in patients with NHL, which possibly due to a relatively low cumulative dose of cyclophosphamide. Also, guidelines on fertility preservation in cancer patients published by American Society of Clinical Oncology classified CHOP (4-6 cycles) as lower risk (<20%) of female gonad toxicity.^[16]^ Cryopreservation of oocytes, embryos or ovarian tissue can be performed before the initiation of treatment for fertility preservation in women. The use of GnRH analogue in combination with chemotherapy may possibly have a protective effect on oocytes.^[17]^ Premature ovarian insufficiency correlates with age, cumulative dose and time schedule of the alkylating agents. Dann et al^[15]^ suggests that increasing the dose of cyclophosphamide only, administered during a short period of time, may help reduce gonadal toxicity in the treatment of aggressive NHL.

Pregnancy and tumorigenesis have some similar events such as immunologic tolerance, angiogenesis, and escaping or editing the host immune response. Little is known about the relationship between these events in pregnancy and in tumor progression and the risk of transmission of maternal cancer to offspring. However, we suspect that transplacental transmission to the fetus is probably rare because of the placental barrier and the fetal alloimmune response.

In conclusion, this present report is very unique because it is the first case that the patient suffered from both infertility and primary extranodal MZBL of the fallopian tube. Our patient successfully became pregnant with in vitro fertilization-embryo transfer after surgery for MZBL, and remained in good condition. More care should be taken when the newly diagnosed mass is combined with a rather high serum CA-125 level. Although endometriosis and ovarian cancer are more common, lymphoma cannot be ruled out.

## Author contributions

Conceptualization: Yundong Mao, Yi Qian.

Funding acquisition: Yi Qian, Jiayin Liu, Yundong Mao.

Resources: Zhen Hou, Jie Huang, Xiang Ma, Yundong Mao.

Supervision: Wei Xu, Cong Wang, Yundong Mao, Yi Qian.

Writing – original draft: Jie Zhang.

Writing – review & editing: Jie Zhang, Nan Lu, Yundong Mao, Yi Qian.

## Footnotes

* Ann-Arbor.
